# The association between psychological distress, abusive experiences, and help-seeking among people with intimate partner violence

**DOI:** 10.1186/s12889-024-18350-y

**Published:** 2024-04-16

**Authors:** Bohan Zhang, Arkers Wong, Rose E. Constantino, Vivian Hui

**Affiliations:** 1 School of Nursing, The Hong Kong Polytechnic University, Hong Kong, Hong Kong SAR China; 2 Health and Community Systems, School of Nursing, University of Pittsburgh, Pennsylvania, PA USA

**Keywords:** Intimate partner violence, Psychological stress, Help-seeking, Mental health

## Abstract

**Background:**

Intimate partner violence (IPV) is a serious public health problem associated with countless adverse physical and mental health outcomes. It places an enormous economic and public health burden on communities. The aim of this study was to examine the associations between psychological states (such as depression or hopeless) and help-seeking experiences of IPV survivors after experiencing IPV, based on the Allegheny County Health Survey (ACHS).

**Methods:**

Data from 2015 to 2016 Allegheny County Health Survey with *N* = 8,012 adults were analyzed. The 6-item version of the Kessler Psychological Stress Scale, located in Module 11 of the ACHS questionnaire, was used to measure psychological stress in participants. Module 12 of the ACHS questionnaire collected information on participants’ experiences of intimate partner violence and help-seeking in the past 12 months. Descriptive statistical analysis, Pearson’s chi-square or two sample independent t-tests statistical analysis, and multivariate binary logistic regression models were used to analyze the relationship between IPV experience and psychological distress.

**Results:**

A total of 212 of the 8,012 participants had IPV experience, with age, marital status, education, income, and race significantly different from those without IPV experience. The psychological stress of participants feeling hopeless (OR = 2.02, 95% CI = 1.37–2.99), restless or fidgety (OR = 1.83, 95% CI = 1.27–2.65), perceiving everything was an effort (OR = 1.55, 95% CI = 1.08–2.22) and worthless (OR = 1.49, 95% CI = 1.01–2.20) was associated with the IPV experience. Help-seeking behaviors of IPV survivors were associated with psychological distress, such as hopelessness (OR = 6.71, 95% CI = 1.38–32.60).

**Conclusions:**

This study explored the association between IPV experience, help-seeking and psychological distress, and the need to expand community support. It is necessary to implement targeted interventions, enhance training of professionals, and promote the identification of early IPV cases as well as collaboration between healthcare and social support departments to reduce the occurrence of IPV or psychological distress following IPV.

**Supplementary Information:**

The online version contains supplementary material available at 10.1186/s12889-024-18350-y.

## Introduction

Intimate partner violence (IPV) is a serious public health issue that primarily affects 25% of women, which accounted for 20% of all violent crimes in the United States [1, 2]. It can negatively impact IPV survivors and their families physically, psychologically, and financially. IPV is associated with a myriad of poor physical and mental health outcomes, such as hypertension, diabetes [3], HIV/AIDS [4], cardiovascular diseases [5], post-traumatic stress disorder, anxiety, and suicidal ideation [6], which are potentially devastating.

Specifically, in the state of Pennsylvania, 37.1% of women and 30.4% of men, experience either physical, sexual, or psychological violence in their lifetime [7]. IPV survivors often experience negative physical, and emotional impacts, such as acute illnesses like respiratory and urinary tract problems, and chronic disorders like migraines and gastrointestinal diseases [8]. It has been found that women who have experienced IPV are more likely to have poor health such as overweight, substance abuse and eating disorders [9]. In addition to physical health, IPV survivors often report negative mental health outcomes. McManus et al., [10] found that 50% of those who had attempted suicide in the past year had experienced IPV, and 31–84% of IPV survivors had posttraumatic stress disorder [11].

Besides IPV survivors suffering from poor health outcomes, the current state of IPV help-seeking is also challenging. Kanougiya et al. analyzed two national surveys of IPV from 2005 to 2006 and 2015–2016, found that the incidence of physical and emotional IPV increased between the two surveys, however formal or informal help-seeking had declined [12]. According to recent single-day statistics retrieved in 2020, due to lockdown and quarantine policy during the COVID-19 pandemic, only an estimated 2,574 survivors had sought help from the state violence prevention programs, in which 198 (7.6%) help-seeking requests went unmet due to inadequate resources provided [13]. However, compared to previous years, the incidence of IPV increased by more than 1.8 times during COVID-19, with sexual and physical violence rising sharply [14].. The most common sources of help-seeking for IPV survivors come from informal resources such as family or friends, rather than formal resources such as police or medical professionals [15]. Less than 10% of IPV survivors exposed to severe forms of violence reported receiving formal help services [16]. It could be related to fear of embarrassment, stigma, concern about receiving accusations, distrust of the justice system or insufficient resources to seek help from IPV [17]. Therefore, exploring the association between IPV experience, help-seeking experiences, and psychological state is necessary to inform local and state-wide policy tailor-made for the IPV population.

Previous research has examined the negative mental health outcomes of IPV survivors through qualitative and quantitative studies, including representative national surveys. Gilbert et al., [18] used National Intimate Partner Violence Survey (NISVS) data from 2010 to 2012 with (*N* = 411,742) to investigate IPV and health condition among U.S. adults. Their results showed a correlation between the intensity of IPV experienced and a subsequent deterioration in mental health status. Cho et al., [9] analyzed the relationship between physical health outcomes and IPV using the NISVS, with no in-depth exploration of mental health conditions. Similarly, Alroy et al., [19] used New York City Community Health Survey data to analyze the prevalence of IPV, health status and behaviors, which found that adults who had experienced psychosocial IPV had a higher prevalence of not getting the treatment they needed for their mental health problems and perceived poorer health status. However, these studies were cross-sectional surveys and there was no causal analysis between IPV and psychological condition. Hui et al., [20] has examined that IPV survivors of sexual violence experience have higher odds of receiving less emotional support and poor health outcomes based on the 2007 Behavioral Risk Factor Surveillance System (BRFSS) survey. Edwards et al., [21] also explored the association between psychological distress and IPV from the same cohort of the BRFSS survey, and found that 15.4% of women with IPV who reported experiences of physical and sexual behavior suffered serious psychological distress. Given that BRFSS removed IPV and psychological distress modules after 2007, there is a paucity of national or locally representative studies to explore further the association between psychological distress and help-seeking experiences among people with IPV experiences in the past decade. Without addressing this knowledge gap, developing evidence-based community support to address such deficiencies among the IPV population will likely remain uncertain. Therefore, the overarching goal of this study is to explore the association between psychological distress, abusive experiences, and help-seeking among people with IPV based on the 2015 Allegheny County Health Survey.

## Method

### Data source and sampling method

We used data from the Allegheny County Health Survey (ACHS), a cross-sectional health survey conducted from August 2015 through September 2016 among Allegheny County adults ages 18 and older. ACHS conducted double-frame random probability-based sampling of landline and cell phone numbers through the digital direct dialing computer system to obtain repeatable and representative samples in each of the 13 county council districts [22]. A total of 9,007 phone interviews were conducted for the ACHS survey, gathering data on demographic characteristics and accessing a number of health indicators, including health behaviors, health diagnoses, and medication use [22].

In this study, we included 8,012 adults aged 18 years or older as a sample who provided complete information on self-reported psychological distress, self-reported IPV with help-seeking sections, and demographic characteristics. The dependent and independent variables in this study were extracted from the Module 11 and Module 12 of the ACHS study questionnaire, respectively.

### Measurement

#### Demographic variables

Data were extracted through the ACHS questionnaire module 1 Core Section, Section 7 Demographics. Sociodemographic characteristics include age, gender, race, marital status, education, and income level.

#### Kessler Psychological Distress Scale

The 6-item version of the Kessler Psychological Distress Scale (K6) was used to measure psychological distress among participants in Module 11 of the ACHS questionnaire [23]. It is a brief and well-known instrument designed by the Pittsburgh health department to screen for nonspecific psychological distress and for quickly and accurately detecting depression and anxiety in the general population, Cronbach’s alpha of K6 was 0.83 [23, 24]. Due to the excellent performance and high efficiency of the scale, it has been widely used in a number of major global and national surveys [25–27]. Several studies have used the K6 to investigate psychological distress in IPV survivors [21, 28]. K6 consisted of six items describing how participants felt in the last 30 days: (1) nervous, (2) hopeless, (3) restless or fidgety, (4) so depressed that nothing could cheer them up, (5) that everything was an effort, and (6) worthless [29]. Each item was self-scored using a 5-point scale ranging from 1 = “none of the time” to 5 = “all of the time”. To ensure accuracy and analyzability of the study, due to the specific nature of psychological distress, we removed data of participants who chose “unsure/don’t know” (*n* = 83), “refused to answer” (*n* = 38), and missing data “null” (*n* = 656), rather than using mean interpolation. As the main focus of this study was on the presence of psychological stress rather than changes from the granularity of categories, we summarized “none of the time” as “not present”, “a little of the time”, “some of the time”, “most of the time”, and “all of the time” as “present” [20].

### Intimate partner violence

Data on IPV were collected by Module 12 of the ACHS questionnaire, which measured the IPV experiences and help-seeking of participants in the past 12 months [29]. There were three questions in the IPV experience part, which covered fright due to threats of violence, specific behaviors of violence, and unwanted sexual activity: “have you been frightened for the safety of yourself, your family, friends, or pets because of the anger or threats of an intimate partner?”, “has an intimate partner hit, slapped, punched, shoved, hoked, kicked, shaken, or otherwise physically hurt you?”, and “has an intimate partner made you take part in any sexual activity when you did not want to, including touching that made you feel uncomfortable?”. The help-seeking experience part included three questions, “have you sought help from a domestic violence hotline or program?”, “have you sought medical care at an emergency room?” and “have you sought a restraining order?”.

Module 12 Questions were answered with “yes”, “no”, “don’t know/not sure”, and “refused”, except for the first question which was answered with “yes”, “no”, “respondent requested to skip to next topic”, “respondent terminated interview at this point”, “don’t know/ not sure”, and “refused”. To ensure accuracy and analyzability of the study, due to the specific nature of psychological distress, we removed data of participants who chose “respondent requested to skip to next topic” (*n* = 157), “respondent terminated interview at this point” (*n* = 18), “don’t know/ not sure” (*n* = 7), “refused” (*n* = 58), and missing data “null” (*n* = 3), rather than using mean interpolation. We also analyzed the difference between the population of people who refused to answer IPV questions and the population who answered IPV questions. The results were statistically different in age, marital status, and income, the detailed results were provided in Additional file 1.

### Statistics analysis

Descriptive statistical analysis was used to determine frequencies and percentages for each categorical variable. Age was a continuous variable and was shown using mean ± standard deviation (SD) after testing for normality. Based on Pearson’s chi-square or two sample independent t-tests statistical analyses, preliminary insights into the associations/relationships between the independent and dependent variables in this study were found.

The independent variable in this study was IPV and the dependent variable was psychological distress. This study used the multivariate binary logistic regression models to estimate the effect of the independent variable on the binary outcome of psychological distress. The multivariate binary logistic regression models are statistical models for estimating the effect of predictors on dichotomous outcome variables [30]. The results of the multivariate binary logistic regression models were expressed using odds ratios (ORs) and 95% confidence intervals (CIs). Statistical significance is indicated by the two-sided *p* value < 0.05. Analyses were conducted using SPSS 28.0 [31].

### Ethics approval and consent to participate

Permission to access the data was granted by the Institutional Review Board of the University of Pittsburgh. Informed consent was not required because the study used secondary data and did not contain any identifiable information.

## Results

### Participants characteristics

A total of 8,012 participants were included in this study, of which a total of 212 (2.65%) participants had abusive experiences of IPV. The results showed that the mean age of participants with experiences of violence was 41.9 years, with 96 (1.2%) males and 116 (1.4%) females. The overall education level of the all participants in this survey was at the general population level, with 7,783 (97.1%) participants with a high school education or higher. The highest annual household income is $75,000+ (*N* = 2,801, 35%), followed by $50,000–$74,999 (*N* = 1,217, 15.2%). Table 1 provided information about the sociodemographic characteristics of the 8,012 participants. Significant factors in differences in IPV abusive experiences include age (*p* < 0.001), martial status (*p* < 0.001), education (*p* < 0.001), and race (*p* < 0.001).

**Table 1:** Sociodemographic characteristics and descriptive analysis of IPV

	Any IPV (Count/%)		Total (*n* = 8012)	t-test or χ^2^ Statistics
	Yes (*n* = 212)	No (*n* = 7800)		
**Age (years)** Mean ± SD	41.94 ± 16.46	54.54 ± 17.60	54.21 ± 17.69	*p* < 0.001
**Gender**				*p* = 0.321
Male	96 (1.2%)	3266 (40.8%)	3362 (42.0%)	
Female	116 (1.4%)	4534 (56.6%)	4650 (58.0%)	
**Marital Status**				*p* < 0.001
Never married	79 (1.0%)	1532 (19.1%)	1611 (20.1%)	
Married	46 (0.6%)	4067 (50.8%)	4113 (51.3%)	
Divorced	42 (0.5%)	910 (11.4%)	952 (11.9%)	
Widowed	10 (0.1%)	869 (10.8%)	879 (11.0%)	
Separated	22 (0.3%)	153 (1.9%)	175 (2.2%)	
A member of an unmarried couple	13 (0.2%)	269 (3.4%)	282 (3.5%)	
**Education**				*p* < 0.001
Never attended school or only attended kindergarten	0 (0.0%)	12 (0.1%)	12 (0.1%)	
Grades 1 through 8 (Elementary)	1 (0.0%)	26 (0.3%)	27 (0.3%)	
Grades 9 through 11 (Some high school)	14 (0.2%)	176 (2.2%)	190 (2.4%)	
Grade 12 or GED (High school graduate)	62 (0.8%)	1836 (22.9%)	1898 (23.7%)	
College 1 year to 3 years (Some college or technical school)	62 (0.8%)	2086 (26.0%)	2148 (26.8%)	
College 4 years or more (College graduate)	73 (0.9%)	3664 (45.7%)	3737 (46.6%)	
**Income**				*p* < 0.001
Less than $10,000	23 (0.3%)	224 (2.8%)	247 (3.1%)	
$999 − 14,999	14 (0.2%)	264 (3.3%)	278 (3.5%)	
$15,000–19,999	21 (0.3%)	396 (4.9%)	417 (5.2%)	
$20,000–24,999	23 (0.3%)	482 (6.0%)	505 (6.3%)	
$25,000–34,999	20 (0.2%)	717 (8.9%)	737 (9.2%)	
$55,000–49,999	21 (0.3%)	921 (11.5%)	942 (11.8%)	
$50,000–74,999	32 (0.4%)	1185 (14.8%)	1217 (15.2%)	
$75,000+	40 (0.5%)	2761 (34.5%)	2801 (35.0%)	
Don’t know	13 (0.2%)	305 (3.8%)	318 (4.0%)	
Refused	5 (0.1%)	545 (6.8%)	550 (6.9%)	
**Race**				*p* < 0.001
White	148 (1.8%)	6546 (81.7%)	6694 (83.5%)	
Black or African American	47 (0.6%)	898 (11.2%)	945 (11.8%)	
American Indian or Alaska Native	4 (0.0%)	41 (0.5%)	45 (0.6%)	
Asian	0 (0.0%)	37 (0.5%)	37 (0.5%)	
Asian Indian	0 (0.0%)	72 (0.9%)	72 (0.9%)	
Chinese	0 (0.0%)	47 (0.6%)	47 (0.6%)	
Filipino	0 (0.0%)	7 (0.1%)	7 (0.1%)	
Japanese	0 (0.0%)	2 (0.0%)	2 (0.0%)	
Korean	0 (0.0%)	4 (0.0%)	4 (0.0%)	
Vietnamese	2 (0.0%)	4 (0.0%)	6 (0.1%)	
Other Asian	0 (0.0%)	20 (0.2%)	20 (0.2%)	
Pacific Islander	0 (0.0%)	1 (0.0%)	1 (0.0%)	
Samoan	0 (0.0%)	1 (0.0%)	1 (0.0%)	
Other Pacific Islander	1 (0.0%)	0 (0.0%)	1 (0.0%)	
OTHER (SPECIFY)	5 (0.1%)	57 (0.7%)	62 (0.8%)	
DON’T KNOW/NOT SURE	1 (0.0%)	14 (0.2%)	15 (0.2%)	
REFUSED	4 (0.0%)	49 (0.6%)	53 (0.7%)	

### Experiences of psychological distress among people with intimate partner violence

As shown in Table 2, there was a significant difference in psychological distress in people who experienced IPV compared to those who did not experience IPV. During the past 30 days, there were 165 people who felt nervous (*p* < 0.001), 109 people who felt hopelessness (*p* < 0.001), 163 people who felt restless or fidgety (*p* < 0.001), 85 people who felt so depressed that nothing could cheer up (*p* < 0.001), 141 people who felt that everything was an effort (*p* < 0.001), and 82 people who felt worthless (*p* < 0.001).

**Table 2:** Descriptive analysis of IPV and psychological distress

	Any IPV (Count/%)		Total (*n* = 8012)	χ^2^ Statistics
	Yes (*n* = 212)	No (*n* = 7800)		
Feel nervous				*p* < 0.001
Not present	47 (22.2%)	3100 (39.7%)	3147 (39.3%)	
Present	165 (77.8%)	4700 (60.3%)	4865 (60.7%)	
Feel hopeless				*p* < 0.001
Not present	103 (48.6%)	6258 (80.2%)	6361 (79.4%)	
Present	109 (43.7%)	1542 (19.8%)	1651 (20.6%)	
Feel restless or fidgety				*p* < 0.001
Not present	49 (23.1%)	3854 (49.4%)	3903 (48.7%)	
Present	163 (76.9%)	3946 (50.6%)	4109 (51.3%)	
Feel so depressed that nothing could cheer up				*p* < 0.001
Not present	127 (59.9%)	6593 (84.5%)	6720 (83.9%)	
Present	85 (40.1%)	1207 (15.5%)	1292 (16.1%)	
Feel that everything was an effort				*p* < 0.001
Not present	71 (33.5%)	4904 (62.9%)	4975 (62.1%)	
Present	141 (66.5%)	2896 (37.1%)	3037 (37.9%)	
Feel worthless				*p* < 0.001
Not present	130 (61.3%)	6755 (86.6%)	6885 (85.9%)	
Present	82 (38.7%)	1045 (13.4%)	1127 (14.1%)	

### Relationship between psychological distress and abusive experiences among people with intimate partner violence

Binary logistics regression models results showed significantly higher odds ratios to explain the association between IPV experience and psychological distress (Table 3). In general, compared to those who had not experienced IPV, people who had experienced any type of IPV had 2.02 times the odds of presenting hopeless (OR = 2.02, 95% CI = 1.37–2.99), 1.83 times the odds of presenting restless or fidgety (OR = 1.83, 95% CI = 1.27–2.65), and 1.55 times the odds of presenting that everything was an effort (OR = 1.55, 95% CI = 1.08–2.22), and 1.49 times the odds of presented worthless (OR = 1.49, 95% CI = 1.01–2.20). People with experiences of IPV of been frightened for the safety had significantly higher odds of presenting feelings of hopelessness (OR = 2.00, 95% CI = 1.24–3.22), restlessness or fidgeting (OR = 1.63, 95% CI = 1.04–2.55), and everything being an effort (OR = 1.58, 95% CI = 1.01–2.46), compared to people without such violent experiences in relationships. Relative to those unexposed to IPV, survivors of hit, slapped, punched, shoved, choked, kicked, shaken, or otherwise physically hurt violence demonstrated 1.80 times (OR = 1.80, 95% CI = 1.07–3.04), 2.76 times (OR = 2.76, 95% CI = 1.60–4.78), and 2.39 times (OR = 2.39, 95% CI = 1.44–3.98) the odds of experiencing hopeless, restlessness or fidgeting, and worthless. The perceived worthlessness of people who have unwanted sex is 2.47 times the odds than that of people who have not experienced this type of violence (OR = 2.47, 95% CI = 1.07–5.71).

**Table 3:** Binary Logistic regression models analysis between intimate partner violence and psychological distress

	IPV experience				IPV Result		
	IPV (any yes) Odds ratio (OR, 95% CI)	IPV (Frightened) OR (95% CI)	IPV (ever violent) OR (95% CI)	IPV (unwanted sex) OR (95% CI)	IPV (domestic violence hotline) OR (95% CI)	IPV (medical care) OR (95% CI)	IPV (sought a restraining order) OR (95% CI)
Nervous	1.04 (0.72–1.51)	1.20 (0.75–1.92)	1.02 (0.61–1.71)	0.48 (0.22–1.05)	0.96 (0.26–3.60)	0.6 (0.16–2.38)	0.86 (0.31–2.40)
Hopeless	2.02 (1.37–2.99) **	2.00 (1.24–3.22) **	1.80 (1.07–3.04) *	2.42 (0.98–5.97)	2.12 (0.62–7.24)	6.71 (1.38–32.60) *	2.38 (0.83–6.81)
Restless or fidgety	1.83 (1.27–2.65) *	1.63 (1.04–2.55) *	2.76 (1.60–4.78) **	2.20 (0.91–5.30)	9.24 (1.13–75.39) *	6.32 (0.75–53.02)	3.57 (1.11–11.50) *
Depressed	1.19 (0.81–1.75)	1.37 (0.86–2.20)	0.97 (0.59–1.60)	1.42 (0.62–3.23)	2.59 (0.80–8.36)	1.87 (0.55–6.39)	2.22 (0.80–6.20)
Feel that everything was an effort	1.55 (1.08–2.22) *	1.58 (1.01–2.46) *	1.53 (0.94–2.50)	1.80 (0.75–4.28)	1.21 (0.36–4.08)	0.84 (0.22–3.23)	0.95 (0.36–2.52)
Worthless	1.49 (1.01–2.20) *	1.37 (0.86–2.19)	2.39 (1.44–3.98) *	2.47 (1.07–5.71) *	1.60 (0.54–4.75)	2.56 (0.76–8.67)	1.12 (0.42–2.99)

* *p* < 0.05, ** *p* < 0.01

IPV: intimate partner violence

Significant differences were also found in analyzing the relationship between the help-seeking of violence, anger, or threats of intimate partner and psychological distress (Table 3). Compared to those who did not seek help from domestic violence hotlines or program, people who sought help from these services demonstrated 9.24 times the odds of presenting restlessness or fidgeting (OR = 9.24, 95% CI = 1.13–75.39). Among IPV survivors who sought medical care in the emergency room, the odds of presenting with hopelessness were 6.71 times (95% CI = 1.38–32.60) than among survivors who had not been exposed to such services. The perceived restlessness or fidgeting of people who have sought a restraining order is 3.57 times the odds than that of people who have not sought (OR = 3.57, 95% CI = 1.11–11.50).

## Discussion

The World Health Organization [32], the World Psychiatric Association [33], and the U.S. Centers for Disease Control (CDC) [34] have identified IPV as a major target for prevention and intervention due to its high prevalence and significant negative impacts. Because psychological distress can be a sequela of IPV, this is a critical issue for mental health professionals [35]. This study analyzed the relationship between abusive experiences among people with IPV and psychological distress using data from the 2015/16 ACHS with *N* = 8,012 participants. The study reported that the experiences of violence were significantly associated with feelings of psychological stress among survivors, such as hopelessness, restlessness or fidgeting, worthlessness and that everything was an effort.

This study found that people who have IPV experience presented psychological distress such as nervousness, hopelessness, depression, and that everything was an effort. The results of this study were consistent with previous studies that IPV experiences have a direct impact on psychological distress [36–38]. Studies [39, 40] have shown that when trauma stemmed from interpersonal violence in an intimate relationship, it had a greater negative impact on a person’s mental health and may lead to post-traumatic stress symptoms as well as alterations in person’s emotion regulation, interpersonal relationships, and self-perceptions, especially when the violent behaviors were perpetrated by someone the victim trusted. Psychological problems are more prevalent in women with IPV than in the general population, with a weighted mean prevalence of depression of 48% among women experiencing IPV, compared with a lifetime prevalence of depression of 10–20% in the general population [41]. The isolation caused by IPV could weaken individual’s self-esteem, thereby triggering a sense of worthlessness and further deepening the depressive symptoms and anxiety of IPV survivors [41].

In this study, the specific behaviors of IPV included hitting, slapping, punching, shoving, choking, kicking, shaking, or other physical injury. This study found that when people experienced these specific physical IPV, they felt 2.76 times higher odds of presenting psychological stress of restlessness or fidgeting than those who did not have these experiences. And among those who suffered unwanted sexual behaviors, the odds of exhibiting worthlessness were 2.47 times higher relative to people who did not experience such violations. This result was consistent with the findings of Cohen et al. (2022) [42] that identified associations between IPV experiences and psychological distress of worthlessness. The possible explanation the link between IPV and psychological distress was that fear and trauma of the abuser may amplify the psychological effects even beyond the onset of physical violence. The IPV survivors may continue to feel unworthy, helpless, or mentally stressed as this fear continues to be exposed to the abuser’s coercive control, even after the violent incident has ended. This prolonged fear and control may maintain higher levels of psychological stress in IPV survivors compared to non-abused individuals [19]. Simultaneously, barriers to accessing mental health treatment, such as lack of health insurance, low self-esteem, and low self-efficacy, may further increase the psychological stress of people with IPV experience [43].

This study also found that people who sought help from IPV hotline or program after IPV experience had higher levels of hopelessness and restlessness or fidgeting than those who did not seek help. One of the possible reasons for this was that IPV survivors with high psychological distress might have higher odds of seeking help to get support to reduce the negative impacts of IPV, such as lowering mental health challenges and reducing stress [44]. Another reason could be because some feared police involvement and contacting law enforcement increased their psychological stress, anxiety, and self-blame [45]. Or it might be caused by racial prejudice, gender discrimination, oppression or other socio-economic factors after the help has been sought [46]. For example, a study has found that Latino adults who have experienced IPV might face additional barriers when seeking social help, including cultural, socio-economic, and legal barriers [46]. Therefore, it is important to understand the help-seeking behaviors and experiences among survivors from different cultural backgrounds before devising a supportive and helpful help-seeking platform, especially as many IPV survivors face barriers to accessing technology. For example, utilizing an online platform that are anonymous, safe and convenient may contribute to positive help-seeking experiences [47].

IPV is a serious public health issue and a leading cause of nonfatal injuries to people in the United States [48]. Despite its high prevalence and serious health consequences, IPV remains largely underreported. This may be partly attributed to embarrassment and stigma barriers that deter or delay survivors from formal help-seeking. Therefore, psychiatric healthcare providers and mental health professionals should be trained to adopt an interpersonal and holistic approach that involves ethical and humane aspects and is the basis for targeted assistance to victims of violence, with a focus on strengthening and empowering people with IPV experiences, rather than only relieving the pain and treating the symptoms and illnesses resulting from the abuse suffered [49]. After identifying IPV, they should participate in documenting the process, working as a team, referring the case to existing intersectoral networks, and ensuring the protection of legal, human, sexual, and reproductive rights, as well as the principles of non-judgment and respect for individual’s decisions, with a focus on communication and assistance [50].

This study revealed the relationship between IPV experiences, help-seeking experiences, and psychological stress. This study’s finding that not only was IPV experience associated with psychological distress, but that help-seeking after IPV was associated with greater distress is important for future research on potential barriers to help-seeking among marginalized groups, which has been less revealed in previous studies. However, several limitations should be considered to interpret the findings of this study. First, the data was obtained from the ACHS, where the IPV modules were not compulsory to answer, thus resulting in some missing data. Due to the specificity of the psychological distress and IPV experience, we chose to delete the sample data for the missing data rather than using statistical methods for interpolation. It might have reduced statistical power by lowering the sample size available for analysis, and the results needs to be interpreted with caution. Second, uncertainty in observed causality due to lack of control for potential confounding variables. In future studies, controlling for potential confounding variables is important to isolate the effects of IPV on psychological health of victims. Thirdly, because the IPV questionnaire used by the ACHS only covered frightened IPV, physical IPV, unwanted sex and help-seeking experiences, we were not able to analyze psychological IPV, which was also very important. Furthermore, although significant associations were found between IPV and psychological distress, the summarized methods we used, the granularity changes from 5 to dichotomous variables may not fully capture the nuances or clinical levels of mental health symptoms. Finally, the cross-sectional nature of the survey data limited the analysis of temporality between IPV survivors and the psychological distress of help-seeking. As the questions assessed exposure to experiences of IPV and psychological distress, we were unable to establish a causal relationship between psychological distress and help-seeking. Further temporal measurement studies are needed to clarify the relationship between IPV experience, help-seeking and psychological distress.

## Conclusion

In summary, findings indicated that self-reported IPV associated with 1.5-9 times higher odds of psychological distress in a sample of American adults in the Allegheny County. Mental health challenges worsened during the COVID-19 pandemic, and with rising mortality rates and inadequate resource allocation in Pennsylvania, the findings of this study highlight the link between IPV and poor psychological health and the need for expanded community support. Targeted interventions to reduce IPV exposure or enhance post-trauma coping strategies may reduce the occurrence of IPV or post-IPV psychological distress. However, cross-sectional data limited the interpretation of the causal relationship between psychological distress and resource use. Future longitudinal temporal studies could clarify the relationship between psychological distress and help-seeking after IPV.

### Electronic supplementary material

Below is the link to the electronic supplementary material.

Supplementary Material 1

## Data Availability

The datasets analyzed in this study are available from the corresponding author with reasonable request.

## Abbreviations

IPV: Intimate partner violence
ACHS: Allegheny County Health Survey
SD: Standard deviation
ORs: Odds ratios
95% CIs: 95% confidence intervals
CDC: Centers for Disease Control

## References

[1] Black MC, Basile KC, Breiding MJ, Smith SG, Walters ML, Merrick MT, Chen J, Stevens M. The national intimate partner and sexual violence survey: 2010 summary report. Centers for Disease Control and Prevention, National Center for Injury Prevention and Control, Division of Violence prevention; 2011. http://www.cdcgov/violenceprevention/pdf/nisvs_report2010-apdf.

[2] Morgan RE, Truman JL, Criminal. victimization, 2018. Bureau of Justice Statistics. 2019; 845:11–18.

[3] Dolezal T, McCollum D, Callahan M. Hidden costs in health care: The economic impact of violence and abuse. 2009.

[4] Li Y, Marshall CM, Rees HC, Nunez A, Ezeanolue EE, Ehiri JE (2014). Intimate partner violence and HIV infection among women: a systematic review and meta-analysis. J Int AIDS Soc.

[5] Constantino R, Angosta A, Reyes A, Kameg B, Wu L, Cobb J, Hui V, Palompon D, Safadi R, Daibes M (2019). Is intimate Partner Violence a risk factor for Cardiovascular Disease in women? A review of the preponderance of the evidence. Health (United Kingdom).

[6] Devries K, Watts C, Yoshihama M, Kiss L, Schraiber LB, Deyessa N, Heise L, Durand J, Mbwambo J, Jansen H (2011). Violence against women is strongly associated with suicide attempts: evidence from the WHO multi-country study on women’s health and domestic violence against women. Soc Sci Med.

[7] Smith SG, Basile KC, Gilbert LK, Merrick MT, Patel N, Walling M, Jain A. National Intimate Partner and Sexual Violence Survey (NISVS): 2010–2012 state report. National Center for Injury Prevention and Control (US) Division of Violence Prevention https://stacks.cdc.gov/view/cdc/46305. 2017.

[8] Kyle J (2023). Intimate Partner violence. Med Clin North Am.

[9] Cho H, Kim W, Nelson A, Allen J (2023). Intimate Partner Violence Polyvictimization and Health outcomes. Violence against Women.

[10] McManus S, Walby S, Barbosa EC, Appleby L, Brugha T, Bebbington PE, Cook EA, Knipe D (2022). Intimate partner violence, suicidality, and self-harm: a probability sample survey of the general population in England. Lancet Psychiatry.

[11] Dutton MA, Green BL, Kaltman SI, Roesch DM, Zeffiro TA, Krause ED (2006). Intimate partner violence, PTSD, and adverse health outcomes. J Interpers Violence.

[12] Kanougiya S, Sivakami M, Daruwalla N, Osrin D (2022). Prevalence, pattern, and predictors of formal help-seeking for intimate partner violence against women: findings from India’s cross-sectional National Family health Surveys-3 (2005–2006) and 4 (2015–2016). BMC Public Health.

[13] National Network to End Domestic Violence (NNEDV). 15th annual domestic violence counts report [https://www.NNEDV.org/DVCounts]. 2020.

[14] Uzoho IC, Baptiste-Roberts K, Animasahun A, Bronner Y (2023). The impact of COVID-19 pandemic on intimate Partner Violence (IPV) Against Women. Int J Soc Determinants Health Health Serv.

[15] Addington LA (2022). Exploring help seeking patterns for emerging adult victims using the national intimate Partner and sexual violence survey. Violence against Women.

[16] Langton L. August. Use of Victim Service Agencies by Victims of Serious Violent Crime, 1993–2009 US Department of Justice. Bureau of Justice Statistics, Special Report 2011.

[17] Wright EN, Anderson J, Phillips K, Miyamoto S (2022). Help-seeking and barriers to care in intimate Partner sexual violence: a systematic review. Trauma Violence Abuse.

[18] Gilbert LK, Zhang X, Basile KC, Breiding M, Kresnow MJ (2023). Intimate Partner Violence and Health conditions among U.S. adults-national intimate Partner Violence Survey, 2010–2012. J Interpers Violence.

[19] Alroy KA, Wang A, Sanderson M, Gould LH, Stayton C. Psychological and Physical Intimate Partner Violence, Measured by the New York City Community Health Survey - New York City, 2018. J Fam Violence. 2022;1–12. 10.1007/s10896-022-00442-1 PMC9510726 36186740

[20] Hui V, Constantino RE (2021). The association between life satisfaction, emotional support, and perceived health among women who experienced intimate Partner violence (IPV)– 2007 behavioral risk factor surveillance system. BMC Public Health.

[21] Edwards VJ, Black MC, Dhingra S, McKnight-Eily L, Perry GS (2009). Physical and sexual intimate partner violence and reported serious psychological distress in the 2007 BRFSS. Int J Public Health.

[22] Hacker K, Brink L, Jones L, Monroe C. Results from the 2015–2016 Allegheny County Health Survey (ACHS): measuring the health of adult residents. Allegheny County Health Department; 2017.

[23] Kessler RC, Andrews G, Colpe LJ, Hiripi E, Mroczek DK, Normand SL, Walters EE, Zaslavsky AM (2002). Short screening scales to monitor population prevalences and trends in non-specific psychological distress. Psychol Med.

[24] Furukawa TA, Kessler RC, Slade T, Andrews G (2003). The performance of the K6 and K10 screening scales for psychological distress in the Australian National Survey of Mental Health and Well-Being. Psychol Med.

[25] Shon EJ (2020). Measurement equivalence of the Kessler 6 psychological distress scale for Chinese and Korean immigrants. Comparison between younger and older adults. Int J Methods Psychiatr Res.

[26] Kasamatsu H, Tsuchida A, Matsumura K, Hamazaki K, Inadera H (2021). Paternal childcare at 6 months and risk of maternal psychological distress at 1 year after delivery: the Japan Environment and Children’s study (JECS). Eur Psychiatry.

[27] Daly M (2022). Prevalence of psychological distress among working-age adults in the United States, 1999–2018. Am J Public Health.

[28] Santambrogio J, Colmegna F, Biagi E, Caslini M, Di Giacomo E, Stefana A, Dakanalis A, Clerici M (2021). Intimate partner violence (IPV) and associated factors: a cross-sectional study in community psychiatry. Riv Psichiatr.

[29] Hacker K, Brink L, Jones L, Monroe C. Results from the 2015–16 Allegheny County health survey (ACHS). Measuring the health of adult residents. Allegh Cty Health Department. 2017. [https://www.alleghenycounty.us/Health-Department/Resources/Data-and-Reporting/Chronic-Disease-Epidemiology/Allegheny-County-Community-Health-Assessment.aspx].

[30] Asmare AA, Agmas YA (2022). Determinants of coexistence of stunting, wasting, and underweight among children under five years in the Gambia; evidence from 2019/20 Gambian demographic health survey: application of multivariate binary logistic regression model. BMC Public Health.

[31] Levesque R. SPSS programming and data management. A guide for SPSS and SAS Users. 2007;102–115.

[32] World Health Organization (2022). Preventing intimate partner violence improves mental health.

[33] Stewart DE (2006). The International Consensus Statement on women’s Mental Health and the WPA Consensus Statement on interpersonal violence against women. World Psychiatry.

[34] Breiding M, Basile KC, Smith SG, Black MC, Mahendra RR. Intimate partner violence surveillance: Uniform definitions and recommended data elements. Version 2.0. 2015.

[35] Stewart DE, Vigod S, Riazantseva E (2016). New developments in intimate Partner Violence and Management of its Mental Health Sequelae. Curr Psychiatry Rep.

[36] Lagdon S, Armour C, Stringer M. Adult experience of mental health outcomes as a result of intimate partner violence victimisation: a systematic review. Eur J Psychotraumatol. 2014; 5. 10.3402/ejpt.v5.24794 PMC4163751 25279103

[37] Richardson R, Nandi A, Jaswal S, Harper S (2020). The effect of intimate partner violence on women’s mental distress: a prospective cohort study of 3010 rural Indian women. Soc Psychiatry Psychiatr Epidemiol.

[38] Fortin I, Guay S, Lavoie V, Boisvert J-M, Beaudry M (2012). Intimate partner violence and psychological distress among young couples: analysis of the moderating effect of social support. J Fam Viol.

[39] Green BL, Goodman LA, Krupnick JL, Corcoran CB, Petty RM, Stockton P, Stern NM (2000). Outcomes of single versus multiple trauma exposure in a screening sample. J Trauma Stress.

[40] Brewin CR, Cloitre M, Hyland P, Shevlin M, Maercker A, Bryant RA, Humayun A, Jones LM, Kagee A, Rousseau C (2017). A review of current evidence regarding the ICD-11 proposals for diagnosing PTSD and complex PTSD. Clin Psychol Rev.

[41] Matheson FI, Daoud N, Hamilton-Wright S, Borenstein H, Pedersen C, O’Campo P (2015). Where did she go? The Transformation of Self-Esteem, Self-Identity, and Mental Well-being among women who have experienced intimate Partner violence. Womens Health Issues.

[42] Cohen F, Seff I, Ssewamala F, Opobo T, Stark L (2022). Intimate Partner Violence and Mental Health: sex-disaggregated associations among adolescents and young adults in Uganda. J Interpers Violence.

[43] Creech S, Davis K, Howard M, Pearlstein T, Zlotnick C (2012). Psychological/verbal abuse and utilization of mental health care in perinatal women seeking treatment for depression. Arch Womens Ment Health.

[44] Voth Schrag RJ, Ravi K, Robinson S, Schroeder E, Padilla-Medina D (2021). Experiences with help seeking among Non-service-engaged survivors of IPV: survivors’ recommendations for service providers. Violence against Women.

[45] Decker MR, Holliday CN, Hameeduddin Z, Shah R, Miller J, Dantzler J, Goodmark L (2019). You do not think of me as a human being: race and gender inequities intersect to discourage police reporting of violence against women. J Urb Health.

[46] O’Neal EN, Beckman LO (2017). Intersections of race, ethnicity, and gender: reframing knowledge surrounding barriers to Social Services among Latina Intimate Partner Violence Victims. Violence against Women.

[47] Hui V, Eby M, Constantino RE, Lee H, Zelazny J, Chang JC, He D, Lee YJ (2023). Examining the supports and advice that women with intimate Partner Violence Experience received in Online Health communities: text Mining Approach. J Med Internet Res.

[48] Stewart DE, Vigod SN (2019). Update on Mental Health aspects of intimate Partner violence. Med Clin North Am.

[49] Fonseca-Machado Mde O, Monteiro JC, Haas VJ, Abrão AC, Gomes-Sponholz F (2015). Intimate partner violence and anxiety disorders in pregnancy: the importance of vocational training of the nursing staff in facing them. Rev Lat Am Enfermagem.

[50] d’Oliveira AF, Schraiber LB, Hanada H, Durand J (2009). [Comprehensive health (care) services to women in gender violence situation: an alternative to primary health care]. Cien Saude Colet.

