# Supplementary material for: The association between psychological distress, abusive experiences, and help-seeking among people with intimate partner violence
Source: BMC Public Health. 2024 Apr 16;24:1060. doi: 10.1186/s12889-024-18350-y (PMC11020675; doi:10.1186/s12889-024-18350-y)
Supplement: Supplementary file 1 — Supplementary Material 1 [file 12889_2024_18350_MOESM1_ESM.docx]

Table 1 Sociodemographic characteristics and descriptive analysis of responders to IPV questions*

|  | Answer question of IPV (Count/%) | | Total (n=8252) | t-test or χ^2^ Statistics |
| --- | --- | --- | --- | --- |
|  | Yes (n=8194) | No (n=58) |  |  |
| **Age (years)**  Mean ± SD | 54.45±17.81 | 64.52±20.70 | 54.52±17.85 | *p* < 0.001 |
| **Gender** |  |  |  | *p* = 0.287 |
| Male | 3426 (41.8%) | 20 (35.5%) | 3446 (41.8%) |  |
| Female | 4768 (58.2%) | 38 (65.5%) | 4806 (58.2%) |  |
| **Marital Status** |  |  |  | *p* < 0.001 |
| Never married | 4155 (50.7%) | 21 (36.2%) | 4176 (50.6%) |  |
| Married | 973 (11.9%) | 4 (6.9%) | 977 (11.8%) |  |
| Divorced | 956 (11.7%) | 21 (36.2%) | 977 (11.8%) |  |
| Widowed | 179 (2.2%) | 2 (3.4%) | 181 (2.2%) |  |
| Separated | 1615 (19.7%) | 9 (15.5%) | 1624 (19.7%) |  |
| A member of an unmarried couple | 284 (3.5%) | 0 (0.0%) | 284 (3.4%) |  |
| Refused | 32 (0.4%) | 1 (1.7%) | 33 (0.4%) |  |
| **Education** |  |  |  | *p* = 0.057 |
| Never attended school or only attended kindergarten | 3 (0.0%) | 0 (0.0%) | 3 (0.0%) |  |
| Grades 1 through 8 (Elementary) | 27 (0.3%) | 0 (0.0%) | 27 (0.3%) |  |
| Grades 9 through 11 (Some high school) | 196 (2.4%) | 3 (5.2%) | 199 (2.4%) |  |
| Grade 12 or GED (High school graduate) | 1969 (24.0%) | 23 (39.7%) | 1992 (24.1%) |  |
| College 1 year to 3 years (Some college or technical school) | 2190 (26.7%) | 13 (22.4%) | 2203 (26.7%) |  |
| College 4 years or more (College graduate) | 3800 (46.4%) | 19 (32.8%) | 3819 (46.3%) |  |
| Refused | 9 (0.1%) | 0 (0.0%) | 9 (0.1%) |  |
| **Income** |  |  |  | *p* < 0.001 |
| Less than $10,000 | 250 (3.1%) | 2 (3.4%) | 252 (3.1%) |  |
| $999-14,999 | 297 (3.6%) | 5 (8.6%) | 302 (3.7%) |  |
| $15,000-19,999 | 439 (5.4%) | 5 (8.6%) | 444 (5.4%) |  |
| $20,000-24,999 | 522 (6.4%) | 4 (6.9%) | 526 (6.4%) |  |
| $25,000-34,999 | 768 (9.4%) | 7 (12.1%) | 775 (9.4%) |  |
| $55,000-49,999 | 959 (11.7%) | 2 (3.4%) | 961 (11.6%) |  |
| $50,000-74,999 | 1232 (15.0%) | 3 (5.2%) | 1235 (15.0%) |  |
| $75,000+ | 2817 (34.4%) | 10 (17.2%) | 2827 (34.3%) |  |
| Don't know | 335 (4.1%) | 6 (10.3%) | 341 (4.1%) |  |
| Refused | 575 (7.0%) | 14 (24.1%) | 589 (7.1%) |  |
| **Race** |  |  |  | *p* = 0.337 |
| White | 6842 (83.5%) | 51 (87.9%) | 6893 (83.5%) |  |
| Black or African American | 966 (11.8%) | 3 (5.2%) | 969 (11.7%) |  |
| American Indian or Alaska Native | 45 (0.5%) | 0 (0.0%) | 45 (0.5%) |  |
| Asian | 40 (0.5%) | 1 (1.7%) | 41 (0.5%) |  |
| Asian Indian | 74 (0.9%) | 1 (1.7%) | 75 (0.9%) |  |
| Chinese | 49 (0.6%) | 1 (1.7%) | 50 (0.6%) |  |
| Filipino | 7 (0.1%) | 0 (0.0%) | 7 (0.1%) |  |
| Japanese | 3 (0.0%) | 0 (0.0%) | 3 (0.0%) |  |
| Korean | 4 (0.0%) | 0 (0.0%) | 4 (0.0%) |  |
| Vietnamese | 6 (0.1%) | 0 (0.0%) | 6 (0.1%) |  |
| Other Asian | 20 (0.2%) | 0 (0.0%) | 20 (0.2%) |  |
| Pacific Islander | 2 (0.0%) | 0 (0.0%) | 0 (0.0%) |  |
| Samoan | 1 (0.0%) | 0 (0.0%) | 0 (0.0%) |  |
| Other Pacific Islander | 1 (0.0%) | 0 (0.0%) | 1 (0.0%) |  |
| OTHER (SPECIFY) | 65 (0.8%) | 1 (1.7%) | 66 (0.8%) |  |
| DON'T KNOW/NOT SURE | 15 (0.2%) | 0 (0.0%) | 15 (0.2%) |  |
| REFUSED | 54 (0.7%) | 0 (0.0%) | 54 (0.7%) |  |

* Those who answered “respondent requested to skip to next topic” (n=157), “respondent terminated interview at this point” (n=18) and “don’t know/ not sure” (n=7) were not deleted from this table.
